# Foreword

**Published:** 1958-01

**Authors:** Philip Morris


### *Foreword*

BY

SIR PHILIP MORRIS, C.B.E., M.A., LL.D.

Education and training for the healing of the sick is older than most universities and at least as deeply buried in the past as the origins of the university tradition. It was already well established and schools for the purpose had come into existence while the work of the doctor was based almost entirely upon the art and craft of the physician. The methods of medical education were those of apprenticeship and the origins of medical science were a by-product of rather than a foundation for medical practice. Thus when a university movement gained strength and the tendency developed for medical schools to be incorporated in universities they brought with them into the university scene a predominantly professional tradition to meet other schools in the universities which were predominantly academic. They have very largely preserved, even through the rapid changes of the last century, a predominantly professional shape and basis. The medical curriculum has remained a curriculum suitable only for intending doctors and its implicit aim and purpose has been to produce men and women who, having met most of the problems of doctoring, were safe to practise the art of healing.

The predominant authority in the profession and consequently the biggest influence over the medical curriculum and medical qualifications has been the General Medical Council. With its desire fully to safeguard the standards of the profession, it has made recommendations to medical schools which have in fact had almost the force of law. As the scientific basis of medicine has become more extensive and as the foundation of treatment has become more and more the application of scientific discoveries for the improvement of diagnosis and therapy, the curriculum has under these influences become more specialized and more fragmented. As it has seemed necessary to emphasize the development of a speciality by causing and encouraging specialization in consulting practice, so in medical education specialization has been allowed to cause analogous departmentalization both in teaching and in examinations. In a state of affairs in which it is impossible for any able and experienced doctor to be well informed in all the clinical specialities we are still in danger of requiring that the medical student shall at least have some acquaintanceship with all of them. In an understandable desire to ensure that a student is wholly ignorant of nothing a new danger has arisen that he will neither be sufficiently well grounded in anything nor, what is still more important, gain a firm grasp of medical scholarship which will ensure that his education continues as his experience and responsibilities grow.

The need for some fundamental reconsideration of the aim and purpose of the medical curriculum has long been apparent. To undertake such a fundamental reconsideration is now possible because a new policy of the General Medical Council has given to universities a new freedom and with this new freedom the responsibility of thinking out afresh the principles upon which the education of doctors should be based.

The first requirement is that the aim and purpose of medical education should be fearlessly examined and re-assessed. The young doctor cannot possibly be taught everything nor can he hope in his undergraduate days to learn everything. The addition to his education of a year of actual experience in hospital under supervision has made it less necessary that the medical curriculum itself should cater for all the professional techniques of day to day importance in medical practice. It is possible for the aim and purpose of the medical curriculum to be more generously defined so that by a re-organization of medical studies the student has both a real opportunity of finding unity in diversity and also an opportunity to become really well established in at least one important scientific discipline. The layman may suggest but only the profession itself, even in a university, can bring about such a result. The present and the years which lie immediately ahead are the time to do it. Whether the output of medical schools is excessive, as some would have us believe, or not is still a matter for discussion, but it is clear that the education and training of the doctor need not suffer from some of the pressures of expansion which must necessarily affect other university activities. As regards the supply of men and women suitable and fitted to practise medicine, there is for the time being no great problem. Such a state of affairs offers ideal circumstances for fundamental reconsideration followed by progressive re-organization and simplification.

It is fortunate that at such a time Professor C. Bruce Perry should be the President of the Bristol Medico-Chirurgical Society. A beloved consultant of rare experience he has recently had the opportunity of concentrating upon the past, present and future trends of medical education in the United States of America. Of what he has seen there, much of which, as might have been expected, has confirmed what he previously thought, he has made the subject of his Presidential Address. It would indeed be pleasant and agreeable to think that his Presidential Address should at least coincide with, if it does not actually cause, a serious movement towards a reformation in medical education. There is a growing willingness to contemplate changes and perhaps the immediate future may be taken as a good time to devise and introduce some of them.

